# Enjoyment in Sport and Alcohol Use among Adolescents: Examining the Mediating Role of Engagement

**DOI:** 10.3390/children11080977

**Published:** 2024-08-13

**Authors:** Vanina Schmidt, Juan Facundo Corti, Ignacio Celsi, María Julia Raimundi, Isabel Castillo

**Affiliations:** 1 National Scientific and Technical Research Council (CONICET), Buenos Aires C1425FQB, Argentina; vschmidt@psi.uba.ar (V.S.); ignaciocelsi@conicet.gov.ar (I.C.); juliaraimundi@mdp.edu.ar (M.J.R.); 2 Research Institute, Faculty of Psychology, University of Buenos Aires, Buenos Aires C1052AAA, Argentina; jfcorti@psi.uba.ar; 3 Faculty of Psychology and Human Relations, Interamerican Open University (UAI), Buenos Aires C1147AAU, Argentina; 4 Institute of Basic, Applied and Technological Psychology (IPSIBAT), National University of Mar del Plata, Mar del Plata B7603ETK, Argentina; 5 Department of Social Psychology, University of Valencia, 46010 Valencia, Spain

**Keywords:** positive youth development, enjoyment, engagement, alcohol, sport

## Abstract

Background: Alcohol consumption among young people is a significant public health concern. Previous studies have indicated that participation in sports, through the positive experiences it offers, may function as a protective factor against alcohol intake. This study aimed to examine the influence of enjoyment and personal fulfillment on adolescent alcohol use, exploring the role of engagement with sports as a mediating variable. Methods: A total of 370 adolescents (mean age = 15.08; *SD* = 1.48) participated in the study and completed the Alcohol Use Disorder Identification Test, the Enjoyment in Sports Scale, and the Athlete Engagement Questionnaire. Results: The results demonstrated that enjoyment is related to lower alcohol consumption through personal fulfillment and engagement in sporting activity. Conclusions: It is crucial to facilitate positive experiences in sport that promote engagement and generate a sense of personal fulfillment, as these factors may reduce the likelihood of risky alcohol consumption.

## 1. Introduction

Alcohol consumption represents a significant public health concern on a global scale, contributing to a considerable proportion of morbidity and mortality among young people [1,2,3]. This phenomenon is shaped by a complex interplay of factors, including peer influence, social pressure (including marketing), the desire to experiment and belong, the accessibility and availability of alcohol, the family environment, cultural and social norms, and psychological characteristics (such as negative emotional states) [4,5]. In contrast to the evidence from other countries, which indicates a decline in alcohol consumption and a later age of onset [6,7], the opposite is occurring in South America. In recent years, an earlier age of onset (77.1% before the age of 14) and a greater frequency of risk patterns (40% of those who use alcohol) have been recorded in Argentina [8]. The most concerning pattern is the so-called heavy episodic drinking (HED), which involves the consumption of a large quantity of alcohol (exceeding 5 BUs = 60 gr/cc of pure alcohol) in a single occasion or short period (3 to 4 h). Among adolescents, it is the most prevalent drinking pattern and has a notable adverse impact on health [9].

Sport represents an excellent opportunity to promote positive youth development and prevent risky behavior. It can foster physical, psychological, and social well-being through the promotion of values, strengths, and a healthy lifestyle [1,10]. Notwithstanding these benefits, a considerable body of research has indicated that participation in team sports is associated with an increased likelihood of alcohol consumption [11,12,13]. In recent years, there has been growing interest in studying the conditions under which sport can serve as a protective factor against alcohol consumption. Some studies suggest that interest, satisfaction, and intrinsic motivation derived from recreational activities such as sport are associated with greater subjective well-being [14,15], personal fulfillment [16], and happiness [17], which in turn may increase or decrease the likelihood of adopting risky behaviors during adolescence [18].

Self-determination theory (SDT) [19] represents a fundamental framework for the comprehension of positive youth experiences in the context of sport. According to SDT, motivational factors associated with sport influence psychological well-being and health-related behaviors [20], consequently reducing the likelihood of engaging in risky behaviors during adolescence [15,21]. SDT proposes that individuals may exhibit two types of motivation when engaging in any activity: intrinsic motivation, which emphasizes enjoyment, interest, and fun as the primary reasons for participation, and extrinsic motivation, where an individual is driven by external rewards or benefits [19]. The most self-determined forms of motivation were associated with lower levels of alcohol, tobacco, and cannabis consumption. Conversely, extrinsic motivation has been linked to higher levels of substance use [22,23], particularly in terms of frequency and excessive alcohol consumption [24].

The present study examined the experience of enjoyment as a positive affective experience that occurs when an individual engages in an activity for intrinsic reasons, thereby satisfying an important desire, goal, or need [19]. The concept is comprised of two components. The first refers to the experience of positive emotional states, including pleasure, satisfaction, gratification, joy, euphoria, enchantment, and passion. The second component, which may be characterized as a cognitive–motivational type, involves an individual who is fully focused and deeply absorbed in the activity, actively pursuing an objective [25,26]. The experience of enjoyment in turn contributes to the development of human potential, meaning in life, and personal fulfillment [25]. Personal fulfillment is defined as the extent to which an individual experiences gratification from achieving significant aspirations and objectives, thereby deriving a sense of purpose and meaning in life [27]. It has been found that personal fulfillment and related phenomena (e.g., purpose in life, meaning in life) are associated with lower alcohol consumption in young people [28,29,30].

Ryan and Martela [31] argue that intrinsically motivated activities (i.e., pursued for the enjoyment they generate) lead to greater engagement. Engagement is known as one of the most optimal experiences that athletes can achieve while playing their sport [32]. The term is defined as a persistent and positive cognitive–affective process comprising four dimensions: the belief in one’s own capacity to achieve a certain level of performance and goals (confidence), the desire to invest effort and time in the pursuit of important objectives (dedication), the presence of physical, mental, and emotional energy or vitality (vigor), and the feeling of enjoyment in the sporting activity (enthusiasm) [33,34].

Prior research conducted with young athletes (aged 15 to 29), has demonstrated a correlation between enjoyment (specifically the cognitive–motivational component called involvement) [35], engagement [36], and personal fulfillment [30] within the context of sport and lower levels and amounts of alcohol consumption. Nevertheless, the combined impact of these three variables on alcohol consumption among adolescents remains unexplored.

In consideration of the presented evidence, the objective of this study was to gain a deeper understanding of the relationship between the enjoyment, personal fulfillment, and engagement through sports and alcohol consumption during adolescence. Accordingly, this study sought to ascertain whether positive experiences derived from engagement in sports can function as protective factors in reducing the propensity for alcohol consumption in adolescents. Specifically, the study aimed to (a) analyze the level and type of alcohol consumption of adolescents who practice sports considering their sex; (b) explore the relationship between positive experiences with each other and with alcohol consumption; and (c) examine whether engagement in sports and personal fulfillment through this practice mediates the relationship between enjoyment and alcohol consumption.

It is therefore hypothesized that (1) adolescents who engage in sports are likely to consume alcohol, and as occurs in the general population, some of them engage in HED, (2) positive experiences are interrelated, and (3) they are associated with lower alcohol intake. Finally, although studies have found that these phenomena separately are associated with lower alcohol consumption, the mediating role of engagement and personal fulfillment has not been previously studied, and therefore, it is not possible to establish a clear hypothesis on this issue.

## 2. Materials and Methods

### 2.1. Participants

Non-probabilistic convenience sampling was employed. To be included in the study, participants were required to be federated athletes who regularly compete at a team (e.g., basketball, handball, volleyball; 90%) or individual (e.g., swimming, tennis, gymnastics; 10%) sports division level. This athlete definition aligns with Swann’s [37] criteria, which include competitive sports participants who have not necessarily reached elite levels (top divisions/Olympics). A minimum sample size of 300 participants was estimated based on the number of independent variables under study [38]. The final sample was composed of 370 athletes (58.6% male and 41.4% female) aged between 12 and 18 years (Mean_age_ = 15.7; SD = 1.55). Regarding sport experience, 73.4% of the sample had practiced their sport for three or more seasons. The majority of participants (64.8%) reported undertaking three or more training sessions per week, while 88.7% of them competed at least once a week.

### 2.2. Instruments

Enjoyment was assessed with the Enjoyment in Sports Scale (EDID) [26]. The scale is composed of 17 items with a 5-point Likert scale (ranging from “totally disagree” to “totally agree”) representing two subscales: (a) positive affect (eight items)—assesses the degree to which an athlete experiences pleasure, satisfaction, gratification, fun, enchantment, and passion (e.g., “I am passionate about practicing this sport”); and (b) involvement (nine items)—assesses the degree to which an athlete finds themself focused on the activity and “captured” (absorbed) by it, in a state of full concentration and total delivery (e.g., “While playing sports, I am very connected to the situation”). The scale shows excellent validity indicators when used with samples of athletes and high reliability. For the present sample, the internal consistency was 0.90 for the total scale, 0.90 for the positive affect subscale, and 0.93 for the involvement subscale.

Engagement was measured using the Athlete Engagement Questionnaire (AEQ) [32,34]. The AEQ assesses, through 16 items with five response options, the frequency with which the athlete perceives the aspects of engagement: confidence (4 items), dedication (4 items), vigor (4 items), and enthusiasm (4 items). Examples of items are “I believe I can achieve my sports goals”, “I give my all to reach my sports goals”, and “I feel full of energy when I practice this sport”. Previous studies demonstrated the psychometric properties of the scales [32]. For the present sample, the internal consistency for confidence was 0.84, for vigor 0.77, for dedication 0.79, for enthusiasm 0.81, and for the total scale 0.92.

The Personal Fulfillment Sense Scale (PFSS) [16] consists of 6 items that, through a 5-point agreement scale, assess the degree of personal fulfillment that subjects perceive to achieve with the activity. Examples of items are “This activity makes me feel accomplished” and “I feel that this activity gives meaning to my life”. Exploratory factor analysis (EFA) by parallel analysis (PA) using the unweighted least squares (ULS) method showed a unidimensional solution (69% of explained variance) with a good fit to the data (Bartlett’s test χ^2^ = 2640.4, *p* < 0.001; KMO = 0.796, *p* < 0.001). For the present sample, the reliability by alpha was 0.86.

Adolescents’ alcohol consumption was assessed using the short version of the Alcohol Use Disorder Identification Test (AUDIT-C) [39,40]. The test employs a 5-point Likert scale comprising three items to measure the frequency of alcohol consumption and the presence and frequency of HED. To measure the presence of HED, participants are required to indicate the number of standard drinks consumed within a relatively brief period of time (approximately three or four hours). A consumption of more than 4 or 5 standard drinks (equivalent to 60 g of pure alcohol for females and males, respectively), indicates the presence of HED. The AUDIT-C showed high sensitivity and low specificity, a varying optimal cut-off point according to sex, acceptable internal consistency, and high test–retest stability [41].

### 2.3. Procedure

Sports clubs were contacted, and after institutional approval, the participants were informed of the procedure and the aim of the study. Informed consent was obtained from parents (for athletes under 16 years old) and participants. Adolescents completed the questionnaires voluntarily and anonymously during a 20 min period after practice. This study was conducted in accordance with the Declaration of Helsinki and the guidelines of the American Psychological Association (APA) and was approved by the Responsible Conduct in Research Committee of the Faculty of Psychology at the University of Buenos Aires (CEI212020 UBA-11-22). The approval date was 2 November 2022.

### 2.4. Data Analysis

A descriptive analysis of the consumption variables was carried out. A chi-squared test was performed to analyze whether there were any significant differences between the sexes with regard to all three consumption variables. To explore the relationships between the positive experiences in sport, a Pearson correlation matrix was estimated and tested. A series of Student’s *t*-tests were performed to analyze differences in the levels of these experiences according to the level of consumption. For *t*-test, the AUDIT-C variables were dichotomized.

A low-frequency group was formed for the frequency-of-consumption variable based on subjects who reported drinking once a month or less, while a high-frequency group was formed based on subjects who reported regular or frequent consumption (twice a month or more).

For the presence of HED, two distinct groups were formed: one comprising individuals who exhibited no evidence of HED (i.e., scores of fewer than 4 standard drinks for females and fewer than 5 standard drinks for males) and another encompassing those who demonstrated indications of HED (i.e., scores of 4 or 5 standard drinks or more for females and males, respectively).

For the variable frequency of HED, two groups were formed: one comprising subjects who reported binge drinking once a month or less (low frequency), and the other comprising those who reported binge drinking once a week or more (high frequency).

Finally, to analyze the mediation effect of personal fulfillment and engagement on the relationship between enjoyment and alcohol consumption, three structural equation models were used. Direct, indirect, and total effects were estimated and tested following Gana and Broc’s [42] guidelines. All analyses were performed using R 4.1.2 [43].

## 3. Results

The prevalence of alcohol consumption among adolescent athletes was found to be consistent across sexes. Table 1 shows these results and the prevalence of high-frequency alcohol consumption, the presence of HED, and the high frequency of HED. A high percentage (70%) of athletes were consuming alcohol at the time of the survey, nearly 30% exhibited a pattern of HED, and one in ten reported a high frequency of this pattern.

Table 2 shows the relationships between the positive experiences in sport and their subscales: engagement (enthusiasm, dedication, vigor and confidence), enjoyment (involvement and positive affect), and personal fulfillment through sport. The three positive experiences were positively and strongly related to each other, with the strongest correlations observed between enjoyment and engagement and positive affect and personal fulfillment. At the component level, the strongest associations were observed between positive affect and vigor, dedication, and enthusiasm.

Table 3 presents the mean differences in positive experiences in sport between athletes who reported higher levels of alcohol consumption and those who did not. Overall, the results indicated a negative relationship between alcohol consumption and personal fulfillment, engagement, as well as confidence, vigor, and enthusiasm.

Mean differences between presence and absence of HED and low and high frequency of HED are presented in Table 4. Once again, personal fulfillment was negatively associated with presence and frequency of HED. Regarding the engagement components, those who reported presence of HED presented lower levels of confidence, and the vigor levels of those who reported lower frequency of HED were higher.

**Figure 1:**
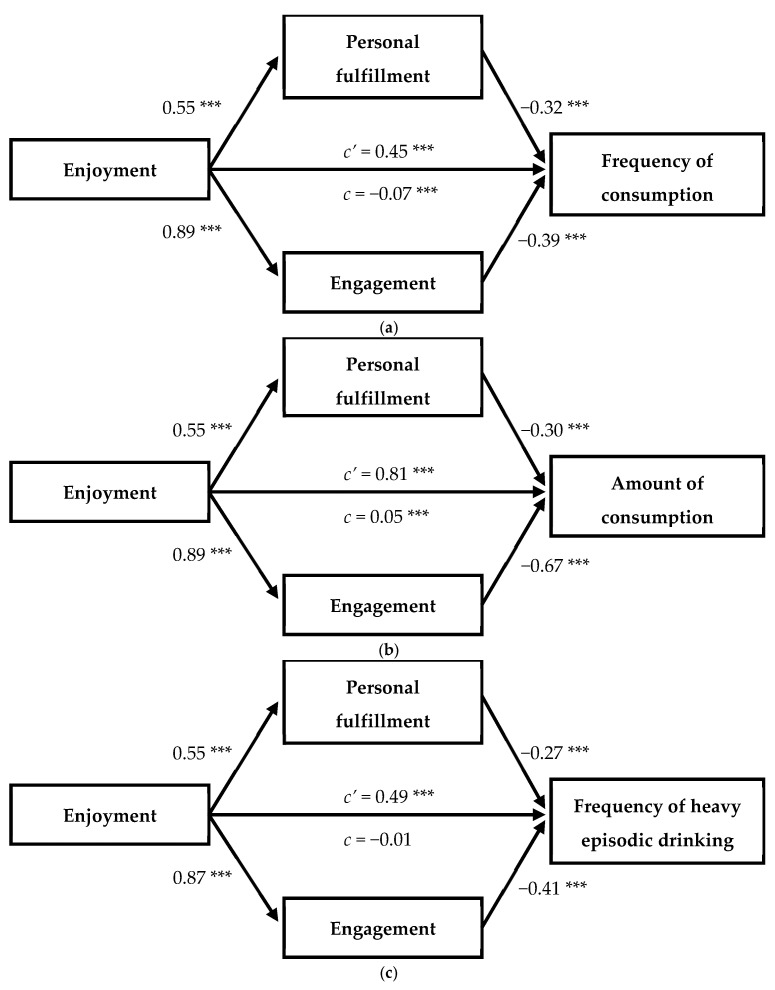
Standardized solution of alcohol consumption prediction through positive experiences in sport: (**a**) frequency of alcohol consumption as criterion; (**b**) amount of alcohol usually consumed per episode; (**c**) frequency of heavy episodic drinking as criterion. *c’* = direct effect, *c* = total effect. *** *p* < 0.001.

The mediation models for predicting alcohol consumption from positive experiences in sport obtained acceptable goodness-of-fit indicators for the frequency of consumption (CFI = 0.971, TLI = 0.952, SRMR = 0.05, *R*^2^ = 0.11), the amount of consumption per episode (CFI = 0.969, TLI = 0.949, SRMR = 0.05, *R*^2^ = 0.16), and the frequency of HED (CFI = 0.964, TLI = 0.940, SRMR = 0.05, *R*^2^ = 0.09). Standardized coefficients of each model are presented in Figure 1. The results indicated that enjoyment in sport had a positive direct effect on alcohol consumption across all three models. However, the total effect of enjoyment on the frequency of consumption was reversed and therefore negative due to the negative indirect effects through personal fulfillment (β = −0.18) and engagement (β = −0.35).

In the frequency of HED model, the total effect of enjoyment was non-significant (indirect effect through personal fulfillment: β = −0.15; indirect effect through engagement: β = −0.36). When considering the amount of alcohol consumed per episode, this effect was positive, but with a significantly smaller effect size than the direct effect (indirect effect through personal fulfillment: β = −0.17; indirect effect through engagement: β = −0.60).

## 4. Discussion

Sport is considered a privileged activity for promoting healthy lifestyles [1], but the literature regarding its relationship with alcohol consumption is inconclusive [11,13]. The results from the present study suggest that a notable proportion of athletes (70%) engage in alcohol consumption despite their regular participation in sports. In adolescents from the general population, the most recent epidemiological study conducted in our country on adolescents [8] revealed that slightly over half (54.7%) reported consuming this substance in the past month. Therefore, it can be concluded that there is a higher prevalence of alcohol consumption among athletes when compared to the general population. Moreover, one-third of adolescent athletes engage in heavy episodic drinking (HED), a rate that is lower in the general population (20%) [9]. Additionally, one in ten adolescents in the sample exhibited this pattern with high frequency. In conclusion, as has been demonstrated in other studies, sports participation is associated with greater alcohol consumption [12,13]. The absence of updated data in our context gives rise to questions as to whether these observed results are specific to the sample of athletes or indicative of an increase in the prevalence of use and risky patterns in recent years. No significant differences were observed in the prevalence of alcohol consumption or the amount of alcohol consumed per occasion when comparing male and female athletes. These results are not unexpected, given that while males have historically reported higher levels of consumption than females, recent decades have witnessed a narrowing of the gender gap, with an increasing number of females consuming more and getting drunk more frequently than their male peers [8].

The robust and unfavorable outcomes observed in this study serve to reinforce the notion that sports participation alone is an insufficient means of deterring the adoption of risky behaviors. One of the reasons for consuming is the search for social interaction [44]. It is plausible that sports, a context conducive to socialization, facilitate gatherings in non-sport settings, which may contribute to elevated levels of consumption. Nevertheless, it is still asserted that sports can serve as a crucial conduit to attaining various sustainable development goals (e.g., health and well-being) among young population groups [1]. However, it is evident that it is necessary to study the experiences associated with sports, not merely participation in the activity [45].

The current study examined three positive experiences: enjoyment, engagement, and personal fulfillment. One of the objectives was to analyze the relationships between these variables and alcohol consumption. A strong relationship was identified between two of the three positive experiences assessed, corroborating the assertion put forth by Ryan and Martela [33] that activities motivated by enjoyment lead to greater engagement. In previous studies [46,47], it was also observed that high levels of self-determined motivation were associated with increased engagement levels in athletes, whereas low levels of self-determined motivation were linked to a decrease in such engagement. The extant literature indicates that the experience of enjoyment and engagement in intrinsically motivated activities contributes to the development of one’s potential and the achievement of personal goals, thereby fostering a sense of personal fulfillment [19,25,31]. The present study lends support to this notion, as it was determined that both enjoyment and engagement are positively, albeit moderately, correlated with a sense of personal fulfillment.

Upon examining the relationship between alcohol consumption and positive experiences associated with sports, it was found that engagement and personal fulfillment were associated with all three variables of the AUDIT. Individuals with higher scores on these variables tended to exhibit lower levels of alcohol consumption. In previous studies, it was found that individuals who presented greater dedication and vigor tended to have lower levels of current alcohol consumption and binge drinking [36]. As evidenced by previous studies [28,29,30], individuals who reported higher levels of personal fulfillment and a sense of purpose in life exhibited lower rates of current alcohol consumption and binge drinking. In the present study, confidence and enthusiasm were observed to be higher among those who consumed alcohol less frequently. Additionally, confidence was found to be higher among those who did not engage in HED, and vigor was observed to be higher among those with low frequency of HED. Similarly, personal fulfillment was associated with a lower frequency of alcohol consumption, absence of HED, and a low frequency of HED. Previous studies [22,23,35] have concluded that intrinsic motivation and enjoyment were associated with lower alcohol and other substance use. However, in the present study, enjoyment was not found to be related to alcohol consumption.

In the mediation models, the role of enjoyment and the other variables is most clearly discernible. All tested mediation models were found to be statistically significant. In these models, enjoyment was found to have a direct positive effect on consumption. The results indicated that increased enjoyment leads to increased consumption. However, it should be noted that enjoyment also increases engagement and personal fulfillment. These two variables exert a negative influence on consumption, resulting in a net negative effect of enjoyment on alcohol consumption. In other words, enjoyment exerts an indirect negative influence on the frequency of consumption through engagement and personal fulfillment. These results suggested that the reduction in consumption is attributable to engagement and personal fulfillment, rather than enjoyment per se. It seems that when enjoyment is the sole motivating factor, the likelihood of alcohol consumption increases. However, when enjoyment fosters engagement and personal fulfillment, it becomes a protective factor, counteracting its potential to act as a risk factor. This dual nature of enjoyment and other hedonic phenomena has been observed in other studies [48], which demonstrates that positive emotions are an essential component of psychological well-being, but can become risk factors when associated with the pursuit of immediate pleasure (which substances, for instance, can provide).

It is important to note that while the effects of some variables on others are discussed, this was a cross-sectional study, which does not allow for the establishment of causal relationships between variables. In future research, a longitudinal design will be used to shed light on the direction of these relationships. Given the legal prohibition of alcohol consumption for individuals under the age of 18, it is possible that the adolescents who participated in the study were not entirely honest in their responses. While strategies such as ensuring confidentiality, anonymity, and voluntary participation can facilitate the collection of truthful answers, it would be prudent to control for potential biases by employing multiple measurement methods. Additionally, future studies should consider whether the sport is individual or team-based. It would also be interesting to include other methods of data collection (such as observation and interviews) in addition to self-report measures.

## 5. Conclusions and Practical Implications

In conclusion, the findings of this study suggest that sports experiences may have a positive impact on the prevention of alcohol consumption, which is currently one of the most significant risk behaviors affecting adolescent health and well-being. Therefore, it is essential to create enjoyable experiences that encourage engagement and foster a sense of personal fulfillment, as these factors may potentially reduce the likelihood of engaging in this behavior.

This study indicates that the quality of sports experiences, rather than the practice itself, should be the focus of attention for health professionals seeking to address the issue of HED and influence public policy. In other words, while promoting access to sports is important, it is also crucial to recognize that they can serve as genuine platforms for engagement and personal fulfillment. As long as sports provide opportunities for these positive experiences, they can contribute to the prevention of alcohol consumption during adolescence, as well as other documented benefits.

As evidenced by the literature [49], the environment (e.g., coaches, team, family) plays a pivotal role in shaping the quality of these experiences. The studies on motivational climate and the coach’s role provide valuable insight into how the environment can foster motivation and sports engagement, thereby contributing to the development of strengths and resources that prevent risky behaviors.

In this sense, the present study provides knowledge that can be relevant for working with these crucial social agents in sport. In the first place, and as the most important figures in the field of sport, this study shows the importance of providing coaches with tools to create environments where engagement is promoted. Through the creation of task-involving climates, autonomy support, and the establishment of learning lines that make adolescents see their progress and improvement, they can favor these positive experiences [50] and thereby protect from alcohol consumption.

In the second place, literature has shown that although coaches play a more important role than parents in this context, the latter still have considerable influence [51]. From this perspective, this work leads us to think that as psychologists, we can provide resources for parents to accompany and encourage their children’s engagement and personal fulfillment through sport. From the circumplex model of marital and family systems, it has been shown that positive family functioning (i.e., high open communication, low communication problems and restricted communication, and very connected and flexible) is not only related to positive experiences in elite youth sport and character strength development [51] but also to less alcohol consumption in adolescents in the general population [52]. Therefore, it is important that sports institutions offer workshops to promote strategies for open communication and support from parents that can foster family contexts that contribute to positive experiences in sport and protect against risky behavior during adolescence.

It is equally important to consider the role of public policies in this network of potential benefits that sports can offer. Such policies should ensure access to recreational sports spaces with leaders who promote quality experiences, especially in vulnerable contexts where health promotion and risk reduction strategies are currently urgently needed.

## Figures and Tables

**Table 1:** Descriptive analysis of alcohol consumption variables and sex differences.

Variables		*n* (%)			*χ*^2^ (df)	*p*
		Global	Female	Male		
Presence of consumption	Absent	107 (29.5)	43 (28.9)	64 (29.9)	0.01 (1)	0.922
	Present	256 (70.5)	106 (71.1)	150 (70.1)		
Frequency of consumption	Low	193 (53.2)	80 (53.7)	113 (52.8)	0.00 (1)	0.952
	High	170 (46.8)	69 (46.3)	101 (47.2)		
Presence of HED	Absent	263 (72.5)	103 (69.1)	160 (74.8)	1.13 (1)	0.288
	Present	100 (27.5)	46 (30.9)	54 (25.2)		
Frequency of HED	Low	322 (88.5)	130 (86.7)	192 (89.7)	0.53 (1)	0.465
	High	42 (11.5)	20 (13.3)	22 (10.3)		

Note. df = degrees of freedom; HED = heavy episodic drinking. For each alcohol consumption variable, the percentages within each sex are presented.

**Table 2:** Bivariate Pearson correlation matrix of the positive experiences in sport.

	1	2	3	4	5	6	7	8
1. Personal fulfillment	-							
2. Positive affect	0.57	-						
3. Involvement	0.15	0.43	-					
4. Confidence	0.28	0.36	0.32	-				
5. Vigor	0.39	0.52	0.37	0.48	-			
6. Dedication	0.45	0.51	0.36	0.51	0.56	-		
7. Enthusiasm	0.39	0.60	0.28	0.40	0.57	0.63	-	
8. Enjoyment	0.37	0.77	0.91	0.39	0.50	0.84	0.48	-
9. Engagement	0.46	0.60	0.41	0.79	0.80	0.49	0.99	0.57

Note. All estimated coefficients were statistically significant (*p* < 0.01).

**Table 3:** Differences in positive experiences in sport between low- and high-frequency consumers.

Criterion	Global	Frequency of Consumption		
		Low	High	*t*
	*M* (*SD*)	*M* (*SD*)	*M* (*SD*)	
Personal fulfillment	3.73 (0.68)	3.89 (0.67)	3.54 (0.64)	5.15 ***
Positive affect	4.60 (0.42)	4.64 (0.41)	4.56 (0.43)	1.95
Involvement	3.82 (0.56)	3.81 (0.55)	3.80 (0.58)	0.16
Confidence	3.73 (0.83)	3.82 (0.83)	3.64 (0.81)	2.01 *
Vigor	4.16 (0.63)	4.22 (0.64)	4.10 (0.61)	1.93
Dedication	4.26 (0.66)	4.33 (0.67)	4.19 (0.66)	1.94
Enthusiasm	4.55 (0.54)	4.60 (0.53)	4.48 (0.55)	2.06 *
Enjoyment	4.19 (0.42)	4.20 (0.40)	4.16 (0.44)	1.02
Engagement	4.18 (0.53)	4.24 (0.54)	4.10 (0.51)	2.49 *

Note. HED = heavy episodic drinking. * *p* < 0.05, *** *p* < 0.001.

**Table 4:** Differences in positive experiences in sport between HED absence and presence and low and high frequency of HED.

Criterion	Presence of HED			Frequency of HED		
	Absent	Present	*t*	Low	High	*t*
	*M* (*SD*)	*M* (*SD*)		*M* (*SD*)	*M* (*SD*)	
Personal fulfillment	3.79 (0.68)	3.58 (0.65)	2.72 **	3.76 (0.68)	3.56 (0.54)	2.18 *
Positive affect	4.61 (0.41)	4.58 (0.43)	0.69	4.60 (0.42)	4.61 (0.42)	−0.09
Involvement	3.80 (0.54)	3.85 (0.61)	−0.68	3.81 (0.53)	3.89 (0.66)	−0.74
Confidence	3.79 (0.82)	3.58 (0.85)	2.08 *	3.74 (0.84)	3.69 (0.79)	0.35
Vigor	4.19 (0.63)	4.09 (0.63)	1.27	4.19 (0.62)	3.95 (0.61)	2.42 *
Dedication	4.27 (0.68)	4.25 (0.63)	0.21	4.27 (0.66)	4.24 (0.64)	0.31
Enthusiasm	4.56 (0.53)	4.52 (0.58)	0.68	4.56 (0.53)	4.51 (0.51)	0.62
Enjoyment	4.18 (0.40)	4.19 (0.46)	−0.17	4.18 (0.40)	4.23 (0.48)	−0.57
Engagement	4.20 (0.54)	4.11 (0.53)	1.44	4.19 (0.53)	4.10 (0.46)	1.20

Note. HED = heavy episodic drinking. * *p* < 0.05, ** *p* < 0.01.

## Data Availability

The data presented in this study are openly available and shared at: http://hdl.handle.net/11336/242308, accessed on 11 August 2024.
